# Retraction: Divergent synthesis of 5-substituted pyrimidine 2′-deoxynucleosides and their incorporation into oligodeoxynucleotides for the survey of uracil DNA glycosylases

**DOI:** 10.1039/d1sc90028e

**Published:** 2021-02-25

**Authors:** Ai Tran, Song Zheng, Dawanna S. White, Alyson M. Curry, Yana Cen

**Affiliations:** Department of Pharmaceutical Sciences, Albany College of Pharmacy and Health Sciences Colchester VT 05446 USA; Department of Medicinal Chemistry, Virginia Commonwealth University Richmond VA 23219 USA ceny2@vcu.edu +1-804-828-7405; Institute for Structural Biology, Drug Discovery and Development, Virginia Commonwealth University Richmond VA 23219 USA

## Abstract

Retraction of ‘Divergent synthesis of 5-substituted pyrimidine 2′-deoxynucleosides and their incorporation into oligodeoxynucleotides for the survey of uracil DNA glycosylases’ by Ai Tran *et al.*, *Chem. Sci.*, 2020, **11**, 11818–11826, DOI: 10.1039/D0SC04161K.

The Royal Society of Chemistry hereby wholly retracts this *Chemical Science* article due to concerns about the reproducibility of the data.

The Royal Society of Chemistry has been contacted by the authors of this article to alert us that recent experiments by their group have shown that some results are not reproducible, especially the yields of several key intermediates. Given the focus on the easy access to epigenetically important nucleosides and related ODNs, unreliable yields significantly impact the confidence in the results. In addition, it was discovered that one of the biological samples provided to the authors was mislabelled. The human stem cells were actually mouse stem cells, hence some discussion and conclusions in the article may no longer apply. Ahead of a thorough re-examination of the entire study, in order to preserve the rigor of the scientific record, the signing authors have chosen to retract the article and they would like to apologise for any inconvenience this may have caused for readers.

Signed: Ai Tran, Song Zheng, Dawanna S. White, Alyson M. Curry and Yana Cen

Retraction endorsed by May Copsey, Executive Editor, *Chemical Science*

## Supplementary Material

